# Complement Inhibition in Severe COVID-19 Acute Respiratory Distress Syndrome

**DOI:** 10.3389/fped.2020.616731

**Published:** 2020-12-29

**Authors:** Sharmila Raghunandan, Cassandra D. Josephson, Hans Verkerke, W. Matthew Linam, Treva C. Ingram, Patricia E. Zerra, Connie M. Arthur, Sean R. Stowell, Michael Briones, Satheesh Chonat

**Affiliations:** ^1^Department of Pediatrics, Emory University School of Medicine, Atlanta, GA, United States; ^2^Aflac Cancer and Blood Disorders Center, Atlanta, GA, United States; ^3^Center for Transfusion and Cellular Therapy, Department of Pathology and Laboratory Medicine, Emory University School of Medicine, Atlanta, GA, United States; ^4^Division of Pediatric Infectious Diseases, Emory University School of Medicine and Children's Healthcare of Atlanta, Atlanta, GA, United States; ^5^Division of Pediatric Intensive Care Unit, Children's Healthcare of Atlanta, Atlanta, GA, United States; ^6^Joint Program in Transfusion Medicine, Department of Pathology, Harvard Medical School, Boston, MA, United States

**Keywords:** complement, COVID - 19, SARS-CoV-2, eculizumab, acute respiratory distress syndrome (ARDS), children, pediatric

## Abstract

Most children with COVID-19 have asymptomatic or mild illness. Those who become critically ill suffer from acute respiratory distress syndrome (ARDS) and acute kidney injury (AKI). The rapid deterioration of lung function has been linked to microangiopathic and immune-mediated processes seen in the lungs of adult patients with COVID-19. The role of complement-mediated acute lung injury is supported by animal models of SARS-CoV, evaluation of lung tissue in those who died from COVID-19 and response of COVID-19 ARDS to complement inhibition. We present a summary of a child with COVID-19 disease treated with convalescent plasma and eculizumab and provide a detailed evaluation of the inflammatory pathways.

## Introduction

Since late 2019 the SARS-CoV-2 virus and resulting COVID-19 illness have spread worldwide, infecting millions and overwhelming health care systems. It is apparent that children and adults are affected in different ways, with children representing <5% of all diagnosed cases, and often asymptomatic or exhibit mild respiratory disease with better prognosis and fewer complications (1). At the same time, it is also now well known that SARS-CoV-2 related multisystem inflammatory syndrome in children (MIS-C) can lead to serious and long-term complications. Though the underlying pathophysiology of this condition is still unclear, its similarities to Kawasaki disease suggests a host immune dysregulation to viral antigens (2). Multiple therapeutic options for severe COVID-19 disease have been tried with varying responses. These include dexamethasone, remdesivir, convalescent plasma (CP), and other immune modulators. ARDS and AKI increase the risk of COVID-19 related mortality (3, 4). COVID-19 associated lung or tissue injury is not directly caused by the SARS-CoV-2 virus, but rather from an aberrant immune response. Once infection occurs, multi-organ failure (MOF) is thought to develop from a combination of endothelial injury, microvascular thrombosis, hypercoagulability, cytokine release, and other inflammatory responses (5, 6). In addition to the existing knowledge on the importance of complement in the host response to viral infections, SARS-CoV infected complement component 3 (C3) knock out mice exhibited a less severe form of ARDS than control mice (7). These mice also demonstrated reduced plasma and intrapulmonary IL-6, the cytokine also implicated in SARS-CoV-2 related inflammatory response (7). This aligns with studies supporting the proposed role of complement activation in ARDS secondary to the generation of anaphylatoxins C3a and C5a, and subsequent neutrophil infiltration (8, 9). Observational studies have uncovered complement activation markers in patients with COVID-19' and eculizumab, a monoclonal antibody against C5, has been reported to improve outcomes in critically ill adults with COVID-19 (6, 10–12). Several trials evaluating the efficacy of complement inhibitors on severe COVID-19 in adults are ongoing. Currently, there is no data evaluating the effectiveness of eculizumab for treating severe COVID-19 in children. This case report describes the use of eculizumab in a teenager with severe COVID-19 disease, and also focuses on complement evaluation that may help guide the management.

## Clinical Description/Results

**Figure 1:**
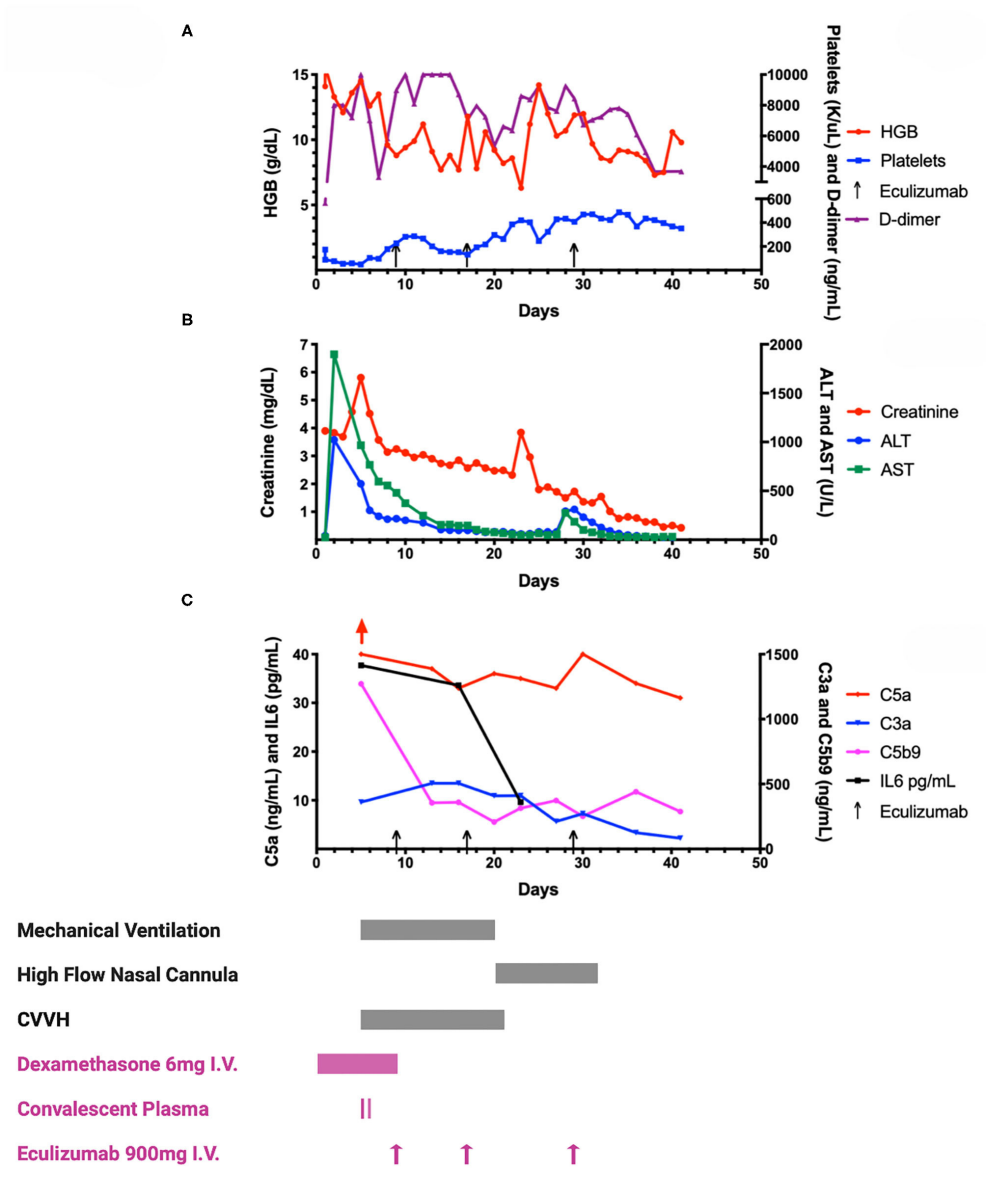
A graphic representation of laboratory and treatment measures. Graphs A-C illustrate various laboratory information throughout the patient's clinical course. Selected clinical interventions (respiratory support, renal replacement therapy, steroids, plasma and eculizumab) are shown along the bottom of this figure, and correspond to the timing of these interventions in relation to the graphs. **(A)** Hemoglobin (HGB), platelet and D-dimer trends throughout hospital course. Patient was admitted to intensive care unit on day 1. As shown in the graph, hemoglobin rapidly trended down along with thrombocytopenia which reached a nadir on day 5 and then steadily recovered following eculizumab and transfusion of platelets. His hemoglobin remained largely stable with a slow decline requiring packed red blood cell transfusions on days 15, 23, and 41. D-dimer was initially 565 ng/mL on day 1 of hospitalization, which rose rapidly over the next few days to peak at 10,000 ng/mL (upper limit of local laboratory analyses). The levels of D-dimer trended downwards around day 30, coinciding with the 3rd dose of eculizumab. The doses of eculizumab are indicated with arrows along the x-axis on days 9, 17, and 29. **(B)** Organ (renal and hepatic) function during COVID-19. Creatinine, Alanine Transaminase (ALT) and Aspartate Transaminase (AST) are shown in relation to the hospital stay of the patient. The peak in creatinine to 5.91 mg/dL on day 5 prompted the use of continuous veno-venous hemofiltration (CVVH) as shown in **(C)**. The fall in creatinine was most appreciated with CVVH, and remained stable during his stay even after CVVH was discontinued on day 22. A brief rise in creatinine was noted after discontinuation of CVVH, which was not sustained, and creatinine levels decreased promptly without any additional intervention except continued eculizumab therapy. Hepatic function steadily improved following convalescent plasma and repeated doses of eculizumab. **(C)** Complement and cytokine analyses during hospital stay. Detailed analyses of multiple markers are in Table 1. This figure depicts the anaphylatoxin complement C5a over 40 ng/mL (red arrow, normal range 2.74–16.33 ng/mL) on day 5 coinciding with respiratory compromise. This level remained high in spite of administration of C5 inhibitor, eculizumab on day 9. The levels started to decline slowly with continue C5 blockade (doses on days 17 and 29). Elevated free C5 level on day 11 (see Table 1), after eculizumab dose on day 9 suggests incomplete blockade of C5 or continued complement activation upstream to C5. Plasma membrane attack complex (C5b9) of 1272 ng/mL which was five times the upper limit of normal (<244 ng/mL) dropped precipitously following eculizumab dose on day 9, and remained well-controlled through the stay. Levels of C3a followed a similar trajectory of C5a. An interesting phenomenon observed was significant drop in interleukin-6 (IL-6) levels following the second dose of eculizumab on day 17, which was closely followed by weaning of mechanical ventilation.

A previously healthy obese 17-year-old African American male presented to the Emergency Department with abdominal pain, nausea and vomiting for 7 days, and a fever of 39.4°C for 4 days. Due to recent contact with his grandfather, who had COVID-19, a nasopharyngeal swab for SARS-CoV-2 by polymerase chain reaction (PCR), was performed and was positive. Over the next 24–36 h, he progressively worsened at home with altered mental status and a seizure-like episode. On arrival to the emergency department, he was noted to have altered mental status and labored breathing. Blood glucose level was 1,861 mg/dL along with severe metabolic acidosis, glycosuria and ketonuria. He was diagnosed with diabetic ketoacidosis secondary to new-onset insulin-dependent diabetes mellitus. The patient responded well to fluid resuscitation and continuous infusion of insulin. Mild respiratory distress and hypoxia in the setting of positive SARS-CoV-2 were initially managed with oxygen by a high-flow oxygen through a nasal cannula (HFNC). Still, he deteriorated quickly, necessitating intubation and mechanical ventilation on 100% oxygen on day 5 of hospital stay (see Figure 1). Supportive care measures on admission included anticoagulation therapy with unfractionated heparin drip. Dexamethasone was initiated at 6 mg intravenously (IV) daily for a total of 10 days for hyper-inflammation and respiratory failure. As shown in Figures 1A–C, he rapidly developed MOF with hepatic dysfunction, pancreatitis, and AKI requiring continuous veno-venous hemofiltration (CVVH). An estimated glomerular filtration rate (eGFR) of <30 and hepatic dysfunction made him ineligible for antiviral therapy with remdesivir. The patient was consented and enrolled on our institutional review board approved expanded access protocol to receive CP on days 5 and 6. The levels of SARS-CoV-2 receptor binding domain (RBD) specific IgG, IgA, and IgM were determined in patient samples and convalescent donor plasma by ELISA using antibody isotype specific detection reagents. The CP provided to the patient on both days were aliquots from the same donor. Antibody endpoint titers (EPT) of patient plasma and CP were determined by estimating the intercept of a sigmoidal fit for a limiting dilution series prepared for each sample with a defined OD cutoff of 0.25. The CP titers from the donor were IgG EPT 20527, IgA EPT 120, and IgM EPT 661. The baseline antibody titers in the patient prior to the first dose of CP were IgG EPT 824, IgA EPT 57, and IgM EPT 70. The RBD specific antibody levels increased (IgG delta EPT +863; IgA EPT +36; IgM delta EPT +62) after the first CP. After a second CP transfusion, the patient's RBD specific IgG titer was observed to increase further by +15127, while IgM and IgA titers remained relatively constant.

**Table 1:** Complement and cytokine analysis during hospitalization with COVID-19.

**Days**	**5,6**	**9**	**11**	**13**	**16**	**17**	**20**	**23**	**27**	**29**	**30**	**34**	**36**	**41**
C3 (83–152 mg/dL)		111												
C4 (13–37 mg/dL)		17												
(Free) C5 (13.5–27 mg/L)			35.9										45.5	
CH50 (38.7–89.9 U/mL)	84					61.3							68.6	
Ba (<1.2 mg/dL)			>8.72										1.8	
Bb (0.49–1.42 mcg/mL)	2.39			4.57	2.79		2.36	2.46	1.72	0.73	1.35		1.74	
C3a (25–88.2 ng/ml)	359.9			>506	>506		409.3	411	212	272.5		126.1	83	88
C3c <2.0 mg/L			2.8											
C5a (2.74–16.33 ng/mL)	>40.0			36.74	33.32		36.49	35.2	33	>40		34	31	
SC5b-9 (≤ 244 ng/mL)	1,272			355	359		208	314	373	252		440	288	
sIL2R (175.3–858.2 pg/mL)	1,140				2,852			1,996						
IF gamma (<4.2 pg/mL)	<4.2				<4.2			<4.2						
IL10 (<2.8 pg/mL)	74				13.4			5.8						
IL1 beta (<6.7 pg/mL)	<6.5				<6.5			<6.5						
IL6 (<2.0 pg/mL)	37.3				33.6			9.6						
IL18 (89–540 pg/mL)	1,019													
CXCL9 (≤ 121 pg/mL)	765													
D-dimer (0–220 ng/mL)	10,000	8,985	8,106	10,000	8,705	7,064	5,338	8,598	7,622	8,434	6,732	7,803	6,099	3,690
CRP (<1 mg/dL)	5.8	10.8		32.3	17.6		13.9	7.4		7.9				7.8
Ferritin (11.1–171.9 ng/mL)	3,914	1,979		1,248	982		856	706		713				562

*C3, complement component 3; C4, complement component 4; free C5, unblocked complement component 5; CH50, screening test for total complement activity; Ba, complement component fragments Ba; Bb, complement component fragments Bb; C3a, complement component fragment 3a; C3c, complement component fragment 3c; C5a, complement component fragment 5a; SC5b-9, soluble membrane attack complex; sIL2R, soluble interleukin 2 receptor; IF gamma, interferon gamma; IL10, Interleukin 10; IL1 beta, Interleukin 1 beta; IL6, Interleukin 6; IL18, Interleukin 18; CXCL9, Chemokine (C-X-C motif) ligand 9; CRP, C-reactive protein. The whole blood samples for complement testing were collected in EDTA anticoagulant tubes and plasma was obtained within 2 h of collection, and stored in −80°C till they were ready for analysis with single thawing. This method of plasma collection in EDTA results in chelation of calcium and magnesium, thus preventing any in vitro complement activation. All testing was obtained in a CLIA certified hospital-based clinical laboratories. All normal values are in parentheses under each value. Top row signifies the laboratory analyses during the hospital stay*.

*On days 9, 17, and 29, the samples were collected prior to administration of eculizumab. Complement proteins C3, C4, and C5 signify the quantitative serum levels. As mentioned in Figure 1C, free C5 level on days 11 and 36 measure the free C5 unbound to eculizumab. Factor B is cleaved to Ba and Bb by factor D in the presence of C3b. Fragment Bb is a serine protease that in combination with hydrolyzed C3 (C3H2O) generates C3 convertase (C3bBb), which augments the cleavage of C3 to generate C3a and C3b. Anaphylatoxins, C3a and C5a are involved in local inflammation and tissue/endothelial damage. C5b-9 contributes to intravascular hemolysis and also deposits on cell membranes. Cytokine panel was significant for elevated IL-6 as seen in COVID-19, suggestive of inflammatory/immune response to the virus and the injury. Elevated sIL2R along with IL10, ferritin and CXCL9 are consistent with inflammation seen in COVID-19*.

As shown in Table 1, the inflammatory profile (c-reactive protein, ferritin, cytokine, and complement pathway markers) were suggestive of COVID-19-related hyperinflammation. By day 7, his respiratory status worsened, demanding increased ventilator settings, and he developed a left pneumothorax and pneumomediastinum. Tocilizumab, a monoclonal antibody directed against the interleukin-6 (IL-6) receptor, has shown benefit in reducing the risk of invasive mechanical ventilation or death from COVID-19 ARDS (13). Though IL-6 was significantly elevated, persistent liver dysfunction precluded its use in our patient.

Given the patient's worsening multiorgan dysfunction, we reasoned that eculizumab use in this critical circumstance was reasonable given its mechanism of action and mounting evidence in complement-mediated ARDS and AKI, (14–16) and other published reports in COVID-19 (6, 10–12). After written consent for the off-label use of eculizumab, the patient was given meningococcal and pneumococcal vaccinations and started on a prophylactic antibiotic regimen. Eculizumab 900 mg IV was initiated on day 9, and subsequent doses on days 17 and 29 were guided by free C5 level and clinical and other laboratory markers (see Table 1). Within 48-h of the first dose of eculizumab, the patient experienced an improvement in respiratory status (decreased pressure support and oxygen requirement) and was extubated on day 20. He was successfully weaned off CVVH by day 22, along with improvement in severe hypertension. Patient has been undergoing rehabilitation with good recovery.

## Discussion

Eculizumab has been used for many years in children with complement disorders such as paroxysmal nocturnal hemoglobinuria, atypical hemolytic uremic syndrome, and transplant associated-thrombotic microangiopathy (TA-TMA) to mitigate hemolysis and organ damage (17–19). Many of these patients are managed by pediatric hematologists and oncologists, who are, therefore, familiar with complement inhibitors. Unlike other complement-mediated TMAs, patients with COVID-19 do not exhibit marked microangiopathic hemolysis. Although we observed a prompt response to platelet count after the 2nd dose of eculizumab in our patient, he did not exhibit evidence of intravascular hemolysis such as schistocytes on blood smear or hemoglobinuria. A recent study reported the potential efficacy of CP in pediatric patients with SARS-CoV-2 ARDS (20). Although CP and dexamethasone may have impacted this patient's outcome by reducing the severity of the disease, the improvement in inflammatory markers following complement inhibition correlated with the improved clinical and respiratory status. Moreover, this improvement was independent of the use of extracorporeal membrane oxygenation, tocilizumab or remdesivir therapy.

The complement pathway was investigated in detail. Total complement activity (CH50) was within normal limits, but elevated anaphylatoxins (C3a, C5a), complement activation degradation product (C3c), and membrane activation complex (C5b9) were consistent with proximal and terminal complement pathway activation. Elevated Bb and Ba confirmed activation of the alternative complement pathway (ACP), which has not been previously shown in COVID-19. This occurs likely secondary to virus mediated activation of the classical or lectin pathways, and formation of C3 degradation product C3b, which drives the ACP. While virus-mediated activation of the classical pathway is a well-known route in viral infections, activation of the lectin pathway by SARS-CoV-2 interaction with mannan-binding lectin-associated serine protease-2 (MASP-2), was initially shown in the lung tissue of patients with COVID-19 (6). Persistently elevated free C5 and C5a levels 2 days after eculizumab suggests COVID-19 related ARDS is associated with marked complement activation, and multiple doses of eculizumab may be required in such cases. We also noticed a remarkable response in interleukin-6 (IL-6) levels following the second dose of eculizumab on day 17, coinciding with weaning of mechanical ventilation to HFNC. These data suggest that while C5b9 may represent terminal complement activation with immediate response to eculizumab, C5a and IL-6 levels together could represent a composite prognostic marker for COVID-19 related ARDS, and could help guide the eculizumab treatment regimen. Rapid improvement in complement profile and IL6 following eculizumab suggests complement-driven endothelial damage may initiate COVID-19 related MOF in some cases. Early administration of eculizumab may hold promise in some patients with severe COVID-19, especially when there is evidence of complement activation. Studies are needed to better understand the mechanisms of complement activation in SARS-CoV-2 infection as well as the efficacy of complement inhibition in COVID-19 related comorbidities such as ARDS, acute kidney injury, thrombosis and multi-organ damage. Additionally, future studies could help explore the synergistic effects of various treatment modalities including complement inhibition, steroids and CP in COVID-19 disease, as our understanding of regulatory host immune responses to the infection continues to grow.

## Data Availability Statement

The original contributions generated for this study are included in the article/supplementary materials, further inquiries can be directed to the corresponding author/s.

## Ethics Statement

Ethical review and approval was not required for the study on human participants in accordance with the local legislation and institutional requirements. Written informed consent to participate in this study was provided by the participants' legal guardian/next of kin. Written informed consent was obtained from the participants' legal guardian/next of kin for the publication of this case report.

## Author Contributions

SR, HV, MB, and SC collected and analyzed the data and wrote the manuscript. CJ, WL, TI, PZ, CA, SS, and SC collected, analyzed the data, and provided critical revisions to the manuscript.

## Conflict of Interest

CJ receives research funds from Terumo BCT, Octapharma and Medtronics. SC is a scientific advisor to Alexion, Novartis and Agios pharmaceuticals. The remaining authors declare that the research was conducted in the absence of any commercial or financial relationships that could be construed as a potential conflict of interest.
